# Identity-by-descent filtering as a tool for the identification of disease alleles in exome sequence data from distant relatives

**DOI:** 10.1186/1753-6561-5-S9-S76

**Published:** 2011-11-29

**Authors:** Nirmala Akula, Sevilla Detera-Wadleigh, Yin Yao Shugart, Michael Nalls, Jo Steele, Francis J McMahon

**Affiliations:** 1 Mood and Anxiety Section, Human Genetics Branch, National Institute of Mental Health, National Institutes of Health, 35 Convent Drive, Bethesda, MD 20892, USA; 2 Unit of Statistical Genomics, National Institute of Mental Health, National Institutes of Health, 35 Convent Drive, Bethesda, MD 20892, USA; 3 Molecular Genetics Section, Laboratory of Neurogenetics, Intramural Research Program, National Institute on Aging, National Institutes of Health, 35 Convent Drive, Bethesda, MD 20892, USA

## Abstract

Large-scale, deep resequencing may be the next logical step in the genetic investigation of common complex diseases. Because each individual is likely to carry many thousands of variants, the identification of causal alleles requires an efficient strategy to reduce the number of candidate variants. Under many genetic models, causal alleles can be expected to reside within identity-by-descent (IBD) regions shared by affected relatives. In distant relatives, IBD regions constitute a small portion of the genome and can thus greatly reduce the search space for causal alleles. However, the effectiveness of this strategy is unknown. We test the simulated mini-exome data set in extended pedigrees provided by Genetic Analysis Workshop 17. At the fourth- and fifth-degree level of relatedness, case-case pairs shared between 1% and 9% of the genome identical by descent. As expected, no genes were shared identical by descent by all case subjects, but 43 genes were shared by many case subjects across at least 50 replicates. We filtered variants in these genes based on population frequency, function, informativeness, and evidence of association using the family-based association test. This analysis highlighted five genes previously implicated in triglyceride, lipid, and cholesterol metabolism. Comparison with the list of true risk alleles revealed that strict IBD filtering followed by association testing of the rarest alleles was the most sensitive strategy. IBD filtering may be a useful strategy for narrowing down the list of candidate variants in exome data, but the optimal degree of relatedness of affected pairs will depend on the genetic architecture of the disease under study.

## Background

Single-nucleotide polymorphism (SNP) microarrays used in genome-wide association studies have been designed to interrogate SNPs with minor allele frequencies (MAFs) greater than or equal to 5%. Genome-wide association studies for a wide variety of complex diseases explain only a small proportion of disease heritability. The so-called missing heritability can be attributed to uncommon and rare variants that are not well interrogated by SNP arrays [1,2]. This observation, combined with major advances in large-scale sequencing methods, has fueled the use of whole-exome and whole-genome sequencing to identify risk variants in common diseases. Using this approach, researchers have successfully identified rare variants involved in Mendelian disorders [3-5], but the number of candidate variants uncovered in these studies has been unexpectedly large, and close to 10,000 variants per individual may be functional. Because common diseases are thought to be genetically heterogeneous [2,6], narrowing down the list of candidate variants to a few causal variants is a challenging process, and the best strategy remains unclear.

To identify loci that encode potential causative alleles, we test the strategy of identity-by-descent (IBD) filtering, that is, isolating IBD regions shared by affected individuals. In distant relatives, IBD regions constitute a small portion of the genome, effectively narrowing the search space for disease alleles under a variety of genetic models [3,6]. IBD analysis may be sufficiently robust to detect loci involved in genetically heterogeneous traits where traditional genetic linkage analysis has failed [3-5,7]. However, the effectiveness of this strategy in the face of high genetic heterogeneity is largely unknown. We apply this strategy to the mini-exome data set of eight large pedigrees in 200 simulated phenotype files provided by Genetic Analysis Workshop 17 (GAW17) (http://www.gaworkshop.org/gaw17/) [8]. When combined with typical filtering and family-based association testing (FBAT), IBD filtering analysis identified five candidate genes that were previously shown to be involved in triglyceride, lipid, and cholesterol metabolism.

## Methods

We analyzed the mini-exome data in the GAW17 family data set, which consists of 697 individuals in eight extended pedigrees. We did not have any knowledge of the actual risk alleles or phenotypes; that is, we did not request the causal genes and markers (answers) from GAW17 until we had completed our analysis.

### Identity by descent

Two or more alleles are identical by descent if they are inherited from the same ancestor. BEAGLE, GERMLINE, and PLINK are some statistical tools that are commonly used to calculate IBD between individuals [9-11], but in the current analysis we use IBD regions provided in the GAW17 simulated data. According to the GAW17 instructions, an IBD score of 0 indicates no sharing, an IBD score of 0.5 indicates sharing of one allele, and an IBD score of 1 indicates sharing of two alleles. However, because without inbreeding only full siblings can share two alleles identical by descent at a locus, an IBD score of 1 does not occur in the GAW17 pedigrees; hence we consider only IBD scores of 0.5 in our analysis.

The percentage of the genome shared (*g*) decreases as the number of meioses (*m*) increases:(1)

First-degree relatives (parent-offspring) share 50% of their genomes, second-degree relatives (grandparents-grandchildren, avuncular pairs) about 25%, third-degree relatives, such as first cousins, about 12.5%, fourth-degree relatives about 6.25%, and fifth-degree relatives about 3.13%. Although these percentages are relatively stable for first-degree relatives, they tend to vary for more distant relatives because of the stochastic nature of recombination events [12].

The first unknown factor involves the optimal degree of relatedness. More closely related cases will likely share more of the same risk alleles but will also share a larger portion of the genome, with many potential variants. More distantly related individuals will share less of the genome but may also carry distinct sets of risk alleles as a result of segregation, the introduction of risk alleles by married-in relatives, and new variants. Because these parameters are generally unknown and because the number of candidate functional variants carried by each individual is large, we opt for a strategy of stringent IBD filtering, focusing on relative pairs who share less than 10% of the genome, corresponding to fourth- and fifth-degree relatives.

**Figure 1:**
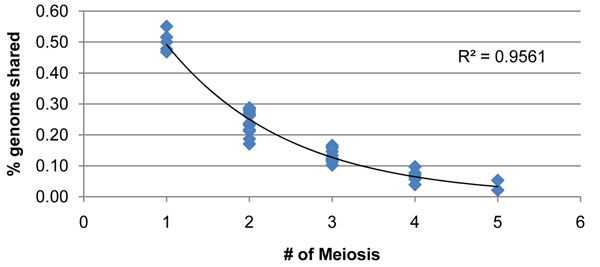
**Fraction of the genome shared between pairs of individuals in pedigree 1 of phenotype file 1 by known number of meioses shown on the pedigree.** The results correspond closely to the expected values of the power function, 1/2*^m^*.

We confirmed the proportion of IBD sharing in the mini-exome data by calculating the total IBD score between all affected pairs of individuals in pedigree 1 of phenotype file 1 (Figure 1). From this analysis, with 95% confidence, we estimated that fourth- and fifth-degree relatives shared between 1% and 9% of the genome. Using these bounds, we selected 95 case-case relative pairs (67 different individuals) in phenotype file 1, excluding any case-case pairs with IBD sharing greater than 10%. We then calculated the number of genes and markers shared by these individuals. The GAW17 mini-exome data consist of 24,488 SNPs in 3,205 genes. We tried to identify SNPs and genes that were shared by all 67 affected individuals in phenotype file 1 but found none.

For each replicate, we ranked genes by the number of case subjects for which the genes had an IBD score greater than 0 (Table 1). We then moved down the list until the number of shared genes fell below 100 and included all those genes in the IBD list for that replicate. We call this “most cases” scoring to distinguish it from “max cases” scoring, in which we selected the gene or genes shared identical by descent by the maximum number of case subjects. As shown in Table 1, 3,205 genes were shared by at least 2 case subjects, 51 genes were shared by up to 31 case subjects (“most cases”), one gene was shared by 34 case subjects (“max cases”), and no genes were shared by more than 34 case subjects. These thresholds were chosen because we could not assume locus homogeneity and wished to minimize the risk of falsely excluding genes that carried true causative alleles. We later evaluated the effects of these thresholds on identifying the true causal genes in the simulated data (see Discussion and Conclusions section).

**Table 1:** IBD filtering in phenotype file 1

Individuals	Genes shared	Total SNPs	Rare SNPs (MAF < 0.1)	Synonymous	Non-synonymous
+2	3,205	24,488	21,605	12,611	8,994
+3	3,193	24,353	21,482	12,528	8,954
+4	3,193	24,353	21,482	12,528	8,954
+5	3,020	23,531	20,730	12,104	8,626
+6	3,018	23,529	20,730	12,104	8,626
+7	2,985	23,336	20,553	11,995	8,558
+8	2,940	23,149	20,383	11,872	8,511
+9	2,924	23,056	20,308	11,830	8,478
+10	2,861	22,852	20,132	11,730	8,402
+11	2,844	22,741	20,035	11,667	8,368
+12	2,635	21,353	18,844	11,026	7,818
+13	2,564	20,914	18,451	10,815	7,636
+14	2,352	19,805	17,550	10,315	7,235
+15	2,221	18,654	16,554	9,731	6,823
+16	2,004	16,837	15,005	8,807	6,198
+17	1,780	15,433	13,748	8,085	5,663
+18	1,665	4,358	12,821	7,551	5,270
+19	1,532	13,125	11,709	6,868	4,841
+20	1,386	12,106	10,786	6,336	4,450
+21	1,236	10,857	9,676	5,681	3,995
+22	1,062	9,332	8,327	4,871	3,456
+23	906	8,133	7,280	4,251	3,029
+24	709	6,470	5,811	3,393	2,418
+25	622	5,520	4,959	2,858	2,101
+26	531	4,705	4,229	2,441	1,788
+27	450	4,352	3,911	2,258	1,653
+28	284	2,857	2,580	1,490	1,090
+29	195	2,090	1,903	1,100	803
+30	120	1,666	1,528	893	635
**+31**	**51**	**867**	**789**	**452**	**337**
+32	24	460	424	249	175
+33	1	16	16	12	4
**+34**	**1**	**16**	**16**	**12**	**4**
+35	0	0	0	0	0

Highlighted **bold** rows indicate the “most cases” and “max cases” respectively.

We repeated this approach for the remaining 199 replicates and then ranked each gene based on the number of replicates in which it was selected [4,5]. Intuitively, this strategy should be quite robust to allelic heterogeneity but less robust to locus heterogeneity. If locus heterogeneity is expected to be high, one could retain genes that overlap with IBD regions in as few as one case-case pair and then use the detected genes from each family as an estimate of the intrafamilial locus heterogeneity.

In summary, our strategy involved the following steps: (1) calculating the IBD score between all pairs; (2) selecting affected pairs; (3) choosing case-case pairs that share between 1% and 9% of the genome; (4) selecting a list of genes shared by most case subjects; (5) repeating steps 1 through 4 for each of the 200 replicate files; (6) ranking each gene based on the number of replicate files from which it was detected.

### Variant filtering

Because the simulated data set was not well suited for sophisticated filtering of variants, we used the commonly applied filters for the MAF in the 1000 Genomes Project data and determined the potential functional impact (nonsynonymous variants). In this case, we applied a 10% MAF threshold, because, in practice, earlier genome-wide association studies could be expected to find more common variants if they conferred reasonable disease risk.

### Family-based association test

To exclude variants that were clearly not associated with the phenotype, we performed an FBAT analysis (http://biosun1.harvard.edu/~fbat/fbat.htm) on all 200 replicate files. We included markers that were informative in at least three out of eight pedigrees. Because there are multiple nuclear families in a pedigree, we used the FBAT option -e, as recommended by the software developer. We then ranked variants by the minimum FBAT *p*-value observed across the 200 replicate analyses. We also performed an FBAT -e analysis after setting the number of informative families to 5 and 8 (8 being the maximum number of pedigrees in the GAW17 data), in order to evaluate the effect of this parameter on the identification of true causal alleles (see Discussion and Conclusions section).

## Results

**Figure 2:**
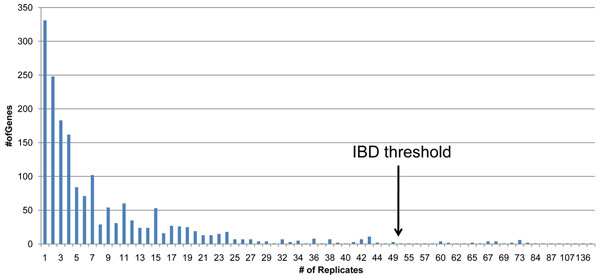
Histogram showing number of genes shared identical by descent by “most cases” across 200 replicates

IBD filtering identified genes that were shared among fourth- and fifth-degree related case subjects in multiple phenotype files. Out of 3,205 genes in the mini-exome data, 1,798 were shared identical by descent by most case subjects in at least one phenotype file. Of these, 43 genes were selected based on sharing by most case subjects in at least 50 phenotype files (Table 2, IBD analysis). The list of 43 genes is shown in Table 3. Figure 2 shows the distribution of IBD sharing across the 200 replicate files.

**Table 2:** Summary of IBD analysis and FBAT analysis

IBD analysis	FBAT analysis
Total genes: 3,205	Total SNPs in 43 genes: 956
Genes shared by most people in at least 1 phenotype file: 1,798	SNPs with < 10% MAF: 876
Genes seen in at least 50 phenotype files: 43	Nonsynonymous SNPs: 525
	FBAT *p* < 0.05: 12

**Table 3:** Top 43 genes from the IBD analysis

Gene	Observed number of phenotype files
F5	140
NF2	136
GC	112
ZNF3	107
TG	88
APOB	87
UGT1A1	86
SH3RF1	84
ADAM29	81
KIAA1712	81
MTERFD2	73
EPHA4	73
HDAC4	73
LOC100129675	73
MYEOV2	73
NDUFA10	73
COL6A3	72
COPS8	72
**VEGFC**	**69**
ACCN4	68
DES	68
SLC4A3	68
TMEM198	68
TTLL4	67
CDH1	67
FN1	67
RNF25	67
TLL1	66
RNMT	65
TKTL2	65
ANP32C	63
LOC100128186	62
NEIL3	61
TMBIM1	61
ERBB4	60
SLC6A3	60
SPHKAP	60
TERT	60
PALLD	58
PLEKHG4B	57
ZNF519	56
XRCC5	55
CA2	53

The **bold** row signifies “VEGFC” a gene that was simulated to be one of the causal genes in GAW17 data.

Variant filtering revealed that the 43 top-ranked genes contained 956 variants. MAF and functional filtering reduced this list to 525 variants in 32 genes (Table 2, FBAT analysis).

Of the 525 variants selected for FBAT analysis, many were seen in only a single family and some were seen in only a few individuals. Although these variants could be true risk alleles, their contribution is impossible to assess in a small sample. Thus we focused on the variants that were seen more frequently in this sample (at least three out of eight families). Of these, 12 variants were associated with the phenotype at a minimum *p*-value less than 0.05 in at least one replicate (Table 2, FBAT analysis). These variants represented five genes: *APOB*, *TTLL4*, *ACCN4*, *COL6A3*, and *TG* (Table 4). The first two columns in Table 4 show the names of the genes followed by the number of replicates in which the genes were selected in the IBD analysis. For example, *APOB* was selected based on case-case sharing in 87 replicates. The remaining columns in Table 4 show the FBAT analysis results for the rare, nonsynonymous variants in those genes that were informative in this data set.

**Table 4:** Candidate genes and variants

IBD analysis		FBAT analysis							
Gene	Number of phenotype files	Marker	MAF	Chromosome	Position	Gene	SNP type	Allele	FBAT *p*-value

*APOB*	87	C2S192	0.079627	2	21078786	*APOB*	Nonsynonymous	T	0.015
*APOB*	87	C2S193	0.041607	2	21078990	*APOB*	Nonsynonymous	G	0.047
*TTLL4*	67	C2S6005	0.033716	2	219311896	*TTLL4*	Nonsynonymous	A	0.035
*ACCN4*	68	C2S6142	0.008608	2	220105317	*ACCN4*	Nonsynonymous	A	0.031
*COL6A3*	72	C2S7528	0.078192	2	237926760	*COL6A3*	Nonsynonymous	A	0.029
*TG*	88	C8S4237	0.083931	8	133969677	*TG*	Nonsynonymous	A	0.046
*TG*	88	C8S4289	0.01363	8	133979643	*TG*	Nonsynonymous	A	0.030
*TG*	88	C8S4456	0.040172	8	134022858	*TG*	Nonsynonymous	G	0.014
*TG*	88	C8S4457	0.040172	8	134022922	*TG*	Nonsynonymous	C	0.014
*TG*	88	C8S4475	0.012195	8	134030339	*TG*	Nonsynonymous	G	0.033
*TG*	88	C8S4540	0.096844	8	134050942	*TG*	Nonsynonymous	C	0.041
*TG*	88	C8S4594	0.02726	8	134093337	*TG*	Nonsynonymous	G	0.044

## Discussion and conclusions

We assume that the GAW17 data set is genetically heterogeneous. Therefore not all affected individuals share the same causal genes (locus heterogeneity), nor do they share the same variants (allelic heterogeneity). We addressed the locus heterogeneity problem by using IBD analysis between distantly related case subjects, selecting genes that were often but not always shared by case subjects. To address allelic heterogeneity, we considered all variants that passed our frequency and functionality filters and all variants located in genes selected by IBD filtering. Larger sample sizes allowed a more liberal IBD filtering, increasing the robustness of this strategy in the face of locus heterogeneity.

Although the IBD filtering did substantially reduce the candidate gene list, there were still 43 candidate genes with many sequence variants. The top hits of the IBD filtering alone were *F5* (shared by case-case pairs in 140 phenotype files) and *NF2* (136 files). Neither of these genes contained variants that were seen in more than a few case subjects. Thus it was important to work down the list to identify variants that were more frequent in this data set. A larger data set would have allowed more discovered variants to be included in the analysis, potentially increasing robustness to allelic heterogeneity.

Filtering of variants based on MAF and potential function cut the list in half, but it is not clear whether this filtering method will be ideal for common complex traits. Depending on penetrance, true risk alleles might be fairly common in comparison data sets, especially those consisting of control subjects who have not been screened for the trait of interest. One could set the MAF threshold higher than 10% and exclude variants that are homozygous in a few control subjects, because these might be more likely to produce a recognized phenotype in control subjects. Similar arguments can be made about functionality. In practice, most studies of complex traits aim to include variants with regulatory or splicing effects, which we could not estimate in the GAW17 data set.

Family-based association testing was the final component of our strategy, aimed at eliminating variants (and genes) that were clearly not associated with the phenotype. In real-world data, power analysis would guide the choice of appropriate *p*-value thresholds for the family-based association testing, and candidates would generally be further evaluated in large case-control samples. Because many rare variants are singletons, nominated genes would typically be resequenced in additional case and control subjects to test the hypothesis that the genes harbor additional deleterious variants in case subjects that might not have been observed in the original study. See Krawitz et al. [5] for a successful example of this strategy.

Our analysis nominated a set of five candidate genes, *APOB*, *TTLL4*, *ACCN4*, *COL6A3*, and *TG*, three of which are implicated in cardiovascular disease. Apolipoprotein B (*APOB*) is the main apolipoprotein component of low-density lipoproteins and is known to play a role in atherosclerotic plaque formation [13]. *ACCN4* (amiloride-sensitive cation channel 4) encodes an amiloride-sensitive sodium channel, and amilorides are often prescribed to control heart failure. The extracellular matrix of arteries and the myocardium have high levels of collagen fibers, and *COL6A3* (alpha 3 type VI collagen isoform 5 precursor) encodes one of the alpha chains of collagen that participates in plaque and clot formation [14].

We necessarily used several arbitrary thresholds in this exercise. Ideally, the optimal thresholds would be selected at the start of a sequencing experiment, guided by the available sample size, replication resources, and educated guesses about the genetic architecture of the disease target.

At the conclusion of the GAW17 meeting, we requested the list of true causal genes so that we could assess the effect of our threshold choices on the results. The list of candidate genes identified by our IBD analysis included *VEGFC*, one of the genes simulated to harbor causal alleles in the GAW17 data (Table 3). However, *VEGFC* contained only one variant that was too rare to be informative in our FBAT analysis. A larger data set might allow more discovered variants to be considered, perhaps by grouping within each gene, potentially increasing robustness to allelic heterogeneity.

More generally, as shown in Table 5, strict IBD filtering that selected genes shared by the maximum number of case subjects in each replicate followed by FBAT analysis was the most sensitive strategy. However, the true-positive rate of 4.6% was still disappointing. This highlights the importance of following up any discovery steps with replication testing in much larger samples.

**Table 5:** Effect of IBD filtering and allele frequency thresholds on sensitivity

FBAT -e Min Size	IBD threshold	
	
	“Most cases” (TP%)	“Max cases” (TP%)
3	13/380 (3.4)	7/151 (4.6)
5	11/354 (3.1)	6/146 (4.1)
8	6/240 (2.5)	4/99 (4.0)

Results of our analysis were compared to the list of true causal genes provided after the conclusion of the GAW17 meeting. “Most cases” refers to the more liberal IBD filtering. “Max cases” refers to the strict IBD filtering that includes genes shared by the maximum number of case subjects in each replicate. “Min Size” refers to the minimum number of nuclear families that carried each candidate variant. TP% is the proportion of identified genes that were actually present on the list of true causal genes, corresponding to a typical measure of sensitivity.

These results suggest that IBD filtering is a promising strategy for narrowing down the list of candidate variants in exome data. Although the sensitivity was low in the simulated GAW17 data, IBD filtering should be particularly effective in founder populations where rare disease alleles are more likely to be inherited from a common ancestor. More theoretical work is needed to determine the optimal degree of relatedness at which case-case pairs should be selected and to identify the best strategy for ranking variants in IBD regions for further study. Much will depend on the genetic architecture of the disease under study.

## Competing interests

The authors declare that there are no competing interests.

## Authors’ contributions

NA participated in study design, performed the analysis and drafted the manuscript. SDW, YYS and MN participated in the design of the study and corrected the manuscript. JS helped with the analysis. FJM conceived the study, participated in its design and helped draft the manuscript. All authors read and approved the final manuscript.

## References

[B1] Maher B Personal genomes: the case of the missing heritability Nature 2008 456 18 21 18987709 10.1038/456018a

[B2] Cirulli ET Goldstein DB Uncovering the roles of rare variants in common disease through whole-genome sequencing Nat Rev Genet 2010 11 415 425 10.1038/nrg2779 20479773

[B3] Ng SB Buckingham KJ Lee C Bigham AW Tabor HK Dent KM Huff CD Shannon PT Jabs EW Nickerson DA Exome sequencing identifies the cause of a Mendelian disorder Nat Genet 2010 42 30 35 10.1038/ng.499 19915526 PMC2847889

[B4] Ng SB Bigham AW Buckingham KJ Hannibal MC McMillin MJ Gildersleeve HI Beck AE Tabor HK Cooper GM Mefford HC Exome sequencing identifies *MLL2* mutations as a cause of Kabuki syndrome Nat Genet 2010 42 790 793 10.1038/ng.646 20711175 PMC2930028

[B5] Krawitz PM Schweiger MR Rödelsperger C Marcelis C Kölsch U Meisel C Stephani F Kinoshita T Murakami Y Bauer S Identity-by-descent filtering of exome sequence data identifies *PIGV* mutations in hyperphosphatasia mental retardation syndrome Nat Genet 2010 42 827 829 10.1038/ng.653 20802478

[B6] Dickson SP Wang K Krantz I Hakonarson H Goldstein DB Rare variants create synthetic genome-wide associations PLoS Biol 2010 8 e1000294 10.1371/journal.pbio.1000294 20126254 PMC2811148

[B7] Roach JC Glusman G Smit AF Huff CD Hubley R Shannon PT Rowen L Pant KP Goodman N Bamshad M Analysis of genetic inheritance in a family quartet by whole-genome sequencing Science 2010 328 636 639 10.1126/science.1186802 20220176 PMC3037280

[B8] Almasy LA Dyer TD Peralta JM Kent JW Jr. Charlesworth JC Curran JE Blangero J Genetic Analysis Workshop 17 mini-exome simulation BMC Proc 2011 5 suppl 9 S2 22373155 10.1186/1753-6561-5-S9-S2 PMC3287854

[B9] Browning SR Browning BL High-resolution detection of identity by descent in unrelated individuals Am J Hum Genet 2010 86 526 539 10.1016/j.ajhg.2010.02.021 20303063 PMC2850444

[B10] Gusev A Lowe JK Stoffel M Daly MJ Altshuler D Breslow JL Friedman JM Pe’er I Whole population, genome-wide mapping of hidden relatedness Genome Res 2009 19 318 326 18971310 10.1101/gr.081398.108 PMC2652213

[B11] Purcell S Neale B Todd-Brown K Thomas L Ferreira MA Bender D Maller J Sklar P de Bakker PI Daly MJ PLINK: a tool set for whole-genome association and population-based linkage analysis Am J Hum Genet 2007 81 559 575 10.1086/519795 17701901 PMC1950838

[B12] Hon L Henn BM Macpherson JM Eriksson N Wojcicki A Avey L Saxonov S Mountain JL Discovering distant relatives within a diverse set of populations using DNA segments identical by descent American Society of Human Genetics, 59th annual meeting, 2009, Abstract 596 http://www.ashg.org/2009meeting/abstracts/fulltext/f10169.htm

[B13] McQueen MJ Hawken S Wang X Ounpuu S Sniderman A Probstfield J Steyn K Sanderson JE Hasani M Volkova E Lipids, lipoproteins, and apolipoproteins as risk markers of myocardial infarction in 52 countries (the INTERHEART Study): a case-control study Lancet 2008 372 224 233 10.1016/S0140-6736(08)61076-4 18640459

[B14] Rodriguez-Feo JA Sluijter JP de Kleijn DP Pasterkamp G Modulation of collagen turnover in cardiovascular disease Curr Pharm Des 2005 11 2501 2514 10.2174/1381612054367544 16026303

